# Microprolactinoma (Pituitary Adenoma) as a Cause of Secondary Headache in a Pediatric Patient With Pain and Restricted Mouth Opening: A Case Report

**DOI:** 10.1155/crid/8912262

**Published:** 2026-04-02

**Authors:** Raluca Draghici, Ovidiu Ionut Saracutu, Matteo Pollis, Daniele Manfredini

**Affiliations:** ^1^ Department of Prosthetics, Carol Davila University of Medicine and Pharmacy, Bucharest, Romania, umfcaroldavila.ro; ^2^ Orofacial Pain Unit, School of Dentistry, Department of Medical Biotechnologies, University of Siena, Siena, Italy, unisi.it

**Keywords:** magnetic resonance imaging, microprolactinoma, pain, secondary headache, temporomandibular disorders

## Abstract

**Introduction:**

The aim of this paper is to report a case of a patient coming to the attention of the general dentist for TMD pain, limited mouth opening, and headache, for which the prescription of a blood test and an magnetic resonance imaging (MRI) led to the incidental diagnosis of a microadenoma of the pituitary gland.

**Case Presentation:**

A 15‐year‐old female came to a dental clinic with a chief complaint of pain at the level of the left temporomandibular joint (TMJ) and reduced mouth opening. The pain is ameliorated by nonsteroidal anti‐inflammatory drugs (ibuprofen) and is aggravated during function (e.g., eating hard food and yawning). In general, the pain is worsened by any movement of the mandible. There was no pain in the other joints. The patient also reported headache in the frontotemporal region. If the intensity of the headache is increased, it is accompanied by episodes of vomiting. The MRI revealed the presence of disc displacement without reduction (DDwoR) in the left joint with limited opening and disc displacement with reduction (DDwR) in the right joint. The presence of myofascial pain was attributed to the presence of tendinitis. The blood sample test revealed an almost double level of prolactin (718.28 mU/mL) compared to the physiological one. The MRI also revealed the presence of a lesion with a 4 mm transversal diameter, 3 mm vertical, and 5 mm anteroposterior diameter compatible with a lateral pituitary microadenoma. Thus, the final diagnosis of microprolactinoma was formulated.

**Conclusion:**

This paper reported a case of an incidentally diagnosed microprolactinoma, which comes to the attention of dental practitioners due to the presence of TMD signs and symptoms. In the presence of uncommon symptoms, such as headache, practitioners should be incentivized to perform further diagnostic tests to rule out the presence of other conditions during the differential diagnosis phase.

## 1. Introduction

The American Academy of Orofacial Pain (AAOP) defines temporomandibular disorders (TMDs) as “a collective term that encompasses a variety of clinical issues that affect the masticatory muscles, temporomandibular joints (TMJs), and associated structures, or both.” They represent the most common cause of nonodontogenic pain, even if the prevalence of TMDs largely varies among different studies [1]. In general, myofascial pain with or without limited mouth opening is the most common finding in TMD patients since up to 75% of them report it [2]. Moreover, pain is almost always the main chief complaint of TMD patients seeking care.

The etiology of TMDs has been debated over the last century. In 1934, it was originally described by Costen [3], an otorhinolaryngologist, who thought that the loss of posterior teeth would cause an uncontrolled condylar movement that would lead to the compression of the auriculotemporal nerve. According to scientific evidence, not only do the occlusal features not represent a risk factor for TMD [4, 5], but the etiology is currently explained at best by the biopsychosocial model [6–8]. A combination of psychological distress [9–12] and mechanical overload of the TMJ due to an excessive frequency of masticatory muscle activities [13, 14] is the most accredited cause for the onset of myofascial pain [15, 16].

The diagnosis of TMDs is mostly clinical and based on the anamnesis and the clinical examination aimed at evaluating the presence, origin, location, and intensity of pain and the functional impairment, which can be assessed and/or reported by patients. In a few cases, the anamnesis and the clinical examination alone cannot give enough information to perform a proper differential diagnosis [17], and the clinician might consider it necessary to prescribe a second‐level diagnostic imaging technique [18]. Over the years, different electronic tools have been proposed to diagnose TMDs, but their diagnostic validity has never been proven and their use for clinical purposes is not supported [18–21].

Instead, the best instrumental option is magnetic resonance imaging (MRI), which represents the gold standard for evaluating the TMJ soft tissue structures in cases of differential diagnosis concerns between intra‐ and extracapsular disorders [22]. A comprehensive evaluation should always be performed when an MRI of the TMJ is prescribed, considering that this technique allows a broad view of the adjacent structures. In such scenarios, dentists and orofacial pain specialists could be among the first sentinels to screen for alterations of the maxillofacial soft tissues that are visible at MRI [23] and even intercept the presence of soft tissue masses and abnormal growths or diseases of the central nervous system (e.g., radiologically isolated syndrome) [24–26] visible at the T2‐weighted MRI. As a rule of thumb, an MRI prescription and comprehensive evaluation are required every time a patient presents symptoms that are atypical for TMD alone, suggesting the presence of concurrent diseases. Headache is one of them.

The aim of this paper is to report a case of a patient coming to the attention of the general dentist for TMD pain, limited mouth opening, and headache, for which the prescription of a blood test and an MRI led to the incidental diagnosis of a microadenoma of the pituitary gland [27]. The case was described according to the CARE guidelines for case reports [28, 29].

## 2. Case Presentation

### 2.1. Patient Information

A 15‐year‐old female came to a dental clinic with a chief complaint of pain at the level of the left TMJ and reduced mouth opening. The patient was well‐oriented and cooperative. She is in 9th grade in high school, an “A” student, and she lives in a high school boarding school. 6 months before, she experienced intense stress due to admission to high school. Instead, the pain appeared 2 days before the consultation after a stressful exam (math test).

The patient reported intermittent pain when moving the mandible. The medical history did not reveal any significant information. She had a body mass index of 22.6 kg/m^2^ (height 153 cm and weight 53 kg). She denied any cranial or facial trauma, and she had no allergies or illness. Moreover, she did not report any assumption of drug, alcohol, or smoking habits. The patient described the pain as sudden, dull, and aching (quasi‐constant). On the visual analog scale (VAS), the intensity was 5. There were no other associated symptoms (e.g., burning, stinging, or itching) with pain, but when it increases in intensity, it provokes feelings of suffocation and fear. The pain is ameliorated by nonsteroidal anti‐inflammatory drugs (ibuprofen) and is aggravated during function (e.g., eating hard food and yawning). In general, the pain is worsened by any movement of the mandible. There was no pain in the other joints. The patient also reported headaches; thus, it was decided to administer the SNOOPPPP (Systemic symptoms or signs, Neurological symptoms or signs, Onset, Older, Previous, Progression, Postural, Pregnancy) algorithm [30]. The headache was in the frontotemporal region. The questionnaire revealed the frequency (infrequent: 1 episode/month), the duration (lasting from 30 min‐6 h), the location (bilateral), the quality (pulsatile), and the severity (8/10) of the headache. If the intensity of the headache increases, it is accompanied by episodes of vomiting. There was no photo/phonophobia, nausea, or aura. Her sleep quality was good (EPWORTH sleepiness scale = 2) [31]. Additionally, the patient reported oligomenorrhea.

The family history revealed that her mother has a history of headaches, which was never investigated.
The patient did not report any relevant past interventions.

### 2.2. Clinical Findings

**Figure 1:**
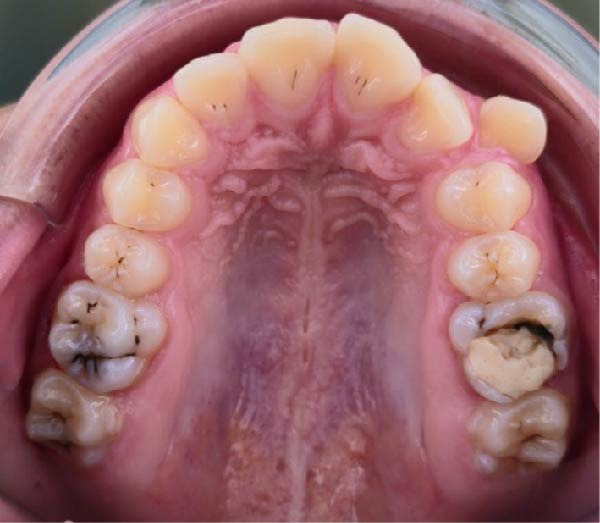
Intraoral picture of the upper arch.

**Figure 2:**
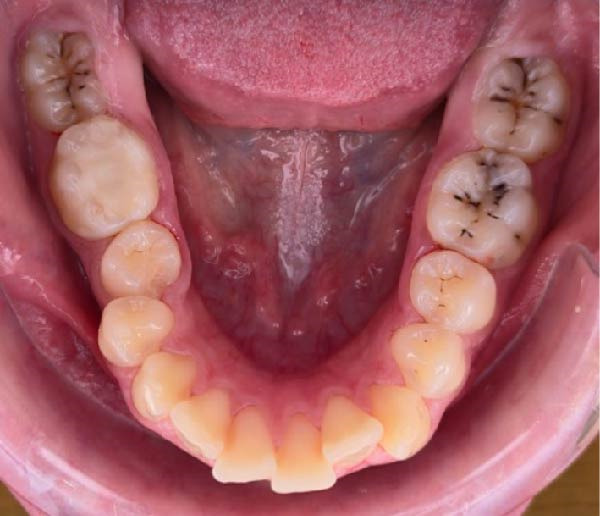
Intraoral picture of the lower arch.

The clinical examination revealed an asymmetry of the upper lip on the left side due to an ectopic upper left canine. The patient presented mild facial hirsutism that scored eight on the Ferriman–Gallwey scale [32]. Intraoral exam revealed a full dentate arch, with four dental caries, asymmetry of the upper dental arch due to an ectopic left canine, deviation of 2 mm of the upper midline to the left, no periodontal problems, and good oral hygiene (Figures 1 and 2). The overbite was 4 mm, while the overjet was 4 mm.

The TMJ function test revealed a range of motion within the normal limits. The patient presented a comfort opening of 30 mm, an active opening of 37 mm, and a passive opening of 40 mm. Mandible movements to the left were 12 mm, to the right were 8 mm, and the protrusion was 8 mm. A click could be heard when opening the right joint, and orthopedic tests on the right increased the pain in the left joint, replicating the familiar pain. To confirm the hypothesis of joint laxity, the examiner started the maneuvers to establish the Beighton Score, and she scored 6/9 [33].

The muscle palpation test started from the caudal to the cranial of the head, with a 0.5‐kg pressure for 5 s for each muscle group. The test revealed mild pain in the cervical area and moderate pain in the deep masseters and bilateral temporalis and trapezius areas with no referral.

Due to the headache history, the cranial nerves were investigated, and everything was within normal limits.

### 2.3. Diagnostic Assessment

Given the role of psychological factors in the etiology of TMDs, the patient was requested to complete a questionnaire for understanding emotional and behavioral disorders: the Romanian version of the translated and validated Depression Anxiety and Stress Scale (DASS 21) [34–36]. The results showed normal scores for depression and anxiety and mild scores for stress [37, 38]. Considering that the patient manifested symptoms that were not exclusively related to TMD, an MRI was also prescribed.

The MRI revealed the presence of disc displacement without reduction (DDwoR) in the left joint with limited opening and disc displacement with reduction (DDwR) in the right joint. The presence of myofascial pain was attributed to the presence of tendinitis. Moreover, the MRI revealed the presence of a lesion in the adenohypophysis.

Considering that the patient reported oligomenorrhea, it was recommended to take additional testing of blood samples. They came back normal, including cortisol, TSH, T3, fT4, ACTH, IGF‐1, LH, FSH, and testosterone/estradiol, except for an elevated prolactin level. Prolactin normal values are normally in the range of 88–484 mU/mL. Her results were almost double, respectively, 718,28 mU/mL.

**Figure 3:**
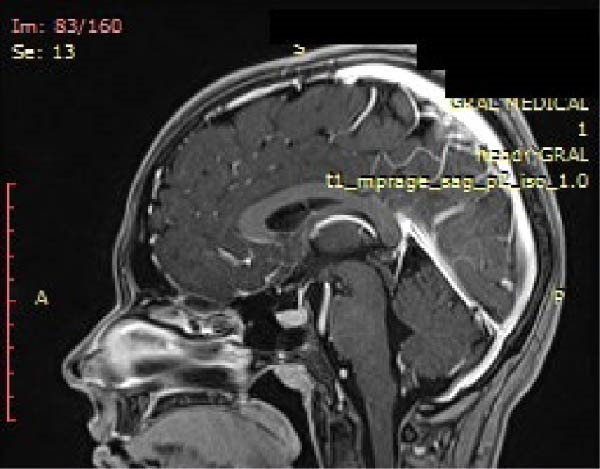
Sagittal T1‐weighted MPRAGE MRI showing a small hypointense lesion within the anterior pituitary gland, consistent with a microprolactinoma. The lesion appears as a subtle and well‐demarcated area disrupting the normal pituitary contour.

**Figure 4:**
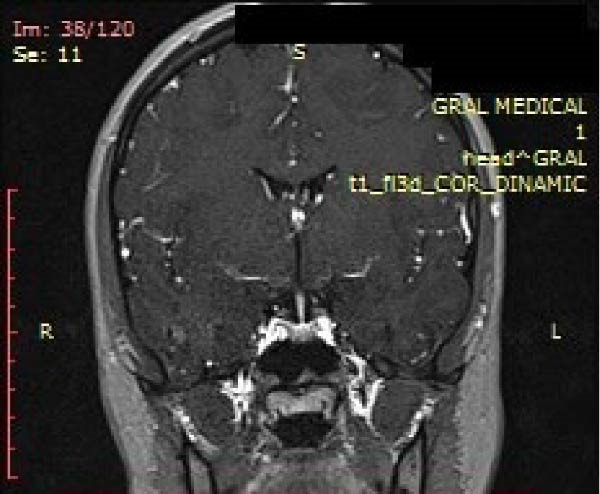
Coronal T1‐weighted postcontrast dynamic MRI showing asymmetric contrast enhancement in the pituitary gland. A small hypointense area on the left side demonstrates delayed enhancement.

**Figure 5:**
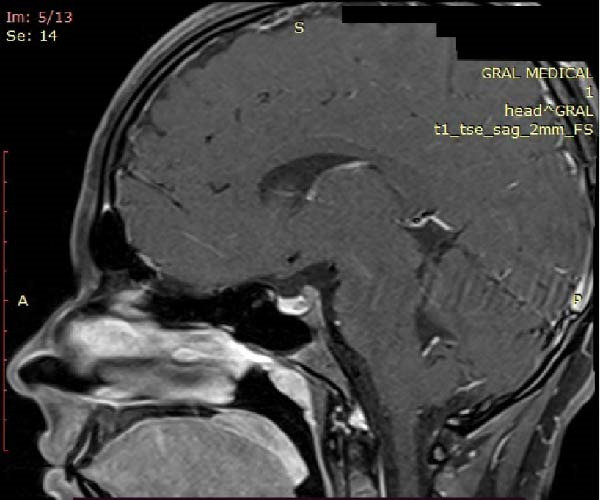
Sagittal T1‐weighted fat‐suppressed post‐contrast MRI showing a well‐defined nonenhancing lesion within the anterior pituitary gland, surrounded by enhancing tissue.

A radiologist specialized in MRI interpretation reviewed the magnetic resonance and described the lesion as a microcode with a 4 mm transversal diameter, 3 mm vertical, and 5 mm anteroposterior diameter compatible with a lateral pituitary microadenoma (Figures 3–5). Based on the present findings, the diagnosis of pituitary gland microprolactinoma was formulated.

According to the International Classification of Headache Disorders, headaches are classified into three categories: primary headaches, secondary headaches, and neuropathies and facial pains [39]. Less than 10% of people with headaches have a secondary headache disorder [40]. In this clinical case, in the first instance, the first hypothetical diagnosis of the primary headache (tension‐type headache or atypically bilateral migraine) was formulated. However, after reviewing the blood sample test and the report from the radiologist, the diagnosis of secondary headache due to prolactinoma hypersecretion was made.

Given the fluctuation and the natural, benign course of TMD, there was no particular concern regarding the evolution of the pain. Considering its low intensity, conservative treatment options are generally enough to prevent symptoms from disappearing [41].

The prognosis of pituitary microprolactinomas is almost always positive, with total remission after 10 years of pharmacological treatment [42]. Compared to macroadenomas, they generally do not cause severe symptoms related to the excessive production of different hormones [43]. The headache should show improvement following the treatment of the microprolactinoma.

### 2.4. Therapeutic Intervention

For the management of the TMD symptoms, the patient was approached with a conservative approach through cognitive behavioral therapy, home exercises, and physiotherapy. It was recommended to postpone orthodontic treatment for the presence of an ectopic canine until the microprolactinoma is in remission.

For treatment and management of the microprolactinoma, the patient was referred to an endocrinologist who proposed the patient’s pharmacologic treatment with a dopamine agonist, cabergoline (Dostinex 0.5 mg, Pfizer, Italy) 0.5 mg/week. Moreover, the patient was referred to a neurologist to see if the treatment of microprolactinoma would decrease the intensity of the headache. In this situation, it should be noted that for the treatment of headache, the administration of tricyclic antidepressants (TCAs; amitriptyline) was postponed until the final diagnosis was reached, considering that amitriptyline is one of the downgrading factors of hyperprolactinemia.

### 2.5. Follow‐Up

The patient was visited at 3 months after the diagnosis. She experienced some adverse symptoms like vomiting and dizziness after the third dose of Dostinex, but in general, the treatment was tolerated. There were still two episodes of headaches.

Following counseling, physiotherapy, and cognitive‐behavioral therapy, the passive mouth opening became 59 mm. The presence of pain at the level of the left TMJ disappeared, while a second click appeared on the opening (at 53 mm), confirming the diagnosis of hypermobility.

The next follow‐up was made 9 months after the diagnosis. She is following the same treatment prescribed by her endocrinologist (Dostinex 0.5 mg, Pfizer, Italy). The patient reports excellent tolerance to the medication, with no adverse symptoms.

The patient experienced the absence of headaches, having no episodes in the last 6 months. This aspect highlights the effectiveness of the treatment in managing symptoms/comorbidities associated with macroprolactinoma.

Additionally, the patient started to understand how to manage the TMD symptoms. Her commitment to prescribed exercises made her able to effectively manage these episodes. She routinely practiced the jaw movement exercises every morning and every time during the day when she feels constricted. Clinically, she exhibits a maximum opening of 54 mm with a click at 33–35 mm during mouth opening with no pain.

## 3. Discussion

The present case report describes the case of a 15‐year‐old female with TMD pain, limited mouth opening, and headache with an associated microprolactinoma of the pituitary gland. Prolactinomas represent less than 2% of all intracranial tumors, and the prevalence of symptomatic macroprolactinoma is approximately 40 per 100,000 individuals [44, 45]. The TMD symptomatology was the chief complaint of the patient who sought dental care. The other clinical symptoms that raised the suspicion of another underlying condition were oligomenorrhea, headache, and hirsutism. The MRI revealed the presence of a lesion at the level of the pituitary gland, which was found to be a microprolactinoma. The blood test showed a marked increase in prolactin, which led to the final diagnosis.

During the anamnesis, the patient reported the presence of stress, which was confirmed by the DASS‐21 questionnaire. Psychological distress and poor copying ability are known to be predisposing factors for TMD due to different pathophysiological mechanisms [46, 47]. Stress increases the odds of somatization and aggravates patients’ perception of pain [15, 48], aspects that may be even more accentuated in females due to biological and hormonal predisposition [49, 50]. On the other hand, psychological factors can predispose individuals to clench and grind their teeth and to brace their mandible [51, 52], increasing the overload to the TMJ structures and leading to subsequent inflammation and functional limitation [13, 53].

Within these premises, it is possible to speculate that treating a patient predisposed to psychological distress due to an ongoing medical condition and undergoing a treatment that by itself was demonstrated to increase anxiety and depression [54] could be more challenging and might require more invasive lines of intervention. Nevertheless, the patient showed complete relief of TMD signs and symptoms already 3 months after the first assessment, in which counseling, physiotherapy, and cognitive‐behavioral therapy were recommended. The moment of receiving a diagnosis of such a rare disease can often have a strong emotional impact on patients [55]. Nonetheless, it is possible to hypothesize that the improvement in TMD symptomatology was positively affected by the counseling process. Informing and reassuring the patient that the conditions that have been diagnosed are not life‐threatening and can be treated with appropriate care and treatment can have a determinant positive impact and improve adherence to therapy [56].

Not surprisingly, the presence of DDwoR on the left side of the joint did not affect the prognosis of the conservative treatment [14]. Unfortunately, still today, the position of the disc, and not the symptomatology of the TMD, is considered the target of many treatment plans that, through surgery and anterior repositioning splints, attempt to restore an ideal condyle‐to‐disc position [57]. Such therapies, besides not having a clinical rationale, have never been proven to be superior to the less invasive options, such as the combination of cognitive behavioral therapy, arthrocentesis [58–60], and the recently proposed botulinum toxin [61].

The positive response to cognitive behavioral therapy and physiotherapy [62] might have also been influenced by the patient’s tolerance towards the pharmacological treatment of macroprolactinomas with dopamine agonists (DAs; Carbegoline), which decreased the level of prolactin. DAs are recommended as the first‐line treatment for prolactinomas to lower the PRL level, reduce tumor size, and restore gonadal function for most patients with prolactinoma [63]. Cabergoline, bromocriptine, and quinagolide are the most important DAs and control most symptoms, but cabergoline has superior efficacy to bromocriptine [64]. Dopaminergic agents are effective and well‐tolerated in children with prolactinoma [65]. In children and adolescents, cabergoline has been used at variable doses (0.5–3.5 mg/week). The frequency of important side effects associated with cabergoline treatment is generally low [66]. Common and temporary side effects may be dizziness, nausea, vomiting, and orthostatic hypotension, which are also caused by the microprolactinoma itself. The treatment’s efficacy and effectiveness are assessed by monitoring the serum prolactin concentrations and tumor size after 6 months. Prompt diagnosis of microprolactinomas and prolactinomas in general is fundamental to avoid complications associated with the disease. In the long term, undiagnosed adolescents develop osteopenia or osteoporosis, conditions that are irreversible even after restoring the normal levels of prolactin through treatment with DAs [67].

The present case is an example of why dentists should not limit their knowledge of nonodontogenic conditions to TMDs and should be aware of the need to screen for different conditions that can be intercepted by administering simple diagnostic tests [68].

## 4. Conclusion

This paper reports a case of an incidentally diagnosed microprolactinoma, which comes to the attention of dental practitioners due to the presence of TMD signs and symptoms. In the presence of uncommon symptoms, such as headache, practitioners should be incentivized to perform further diagnostic tests to rule out the presence of other conditions during the differential diagnosis phase.

## Author Contributions

**Raluca Draghici**: writing – original draft, writing – review and editing, data curation, visualization, investigation, resources. **Ovidiu Ionut Saracutu**: writing – original draft, writing – review and editing, supervision, validation. **Matteo Pollis**: data curation, visualization, validation. **Daniele Manfredini**: conceptualization, supervision, methodology, writing – review and editing, visualization, validation, project administration.

## Funding

The authors reported that there is no funding associated with the work featured in this article.

## Ethics Statement

This case report obtained ethical approval from the Ethical Review Committee of the Siena Orofacial Pain Board (Approval Number IRB‐2024‐381). This case report was prepared following the CARE guidelines.

## Consent

The author confirms that the patient’s mother has given informed consent to participate in the research.

## Conflicts of Interest

The authors declare no conflicts of interest.

## Data Availability

The data that support the findings of this study are available from the corresponding author upon reasonable request.
